# *HER2* amplification by next-generation sequencing to identify *HER2*-positive invasive breast cancer with negative HER2 immunohistochemistry

**DOI:** 10.1186/s12935-022-02761-1

**Published:** 2022-11-15

**Authors:** Laura Morsberger, Aparna Pallavajjala, Patty Long, Melanie Hardy, Rebecca Park, Rebecca Parish, Azin Nozari, Ying S. Zou

**Affiliations:** 1 Department of Pathology, Cancer Genetics Laboratory, Cytogenetics and Genomics Lab, Molecular Diagnostic Lab, JH Genomics, Genomic Medicine and Pathology, Johns Hopkins University School of Medicine, 1812 Ashland Ave., Suite 221, Baltimore, MD 21205 USA; 2 The Johns Hopkins Genomics, Johns Hopkins University School of Medicine, Baltimore, MD USA; 3 Cytogenetics Laboratory, Johns Hopkins University Hospital, Baltimore, MD 21205 USA

**Keywords:** Breast cancer, Fluorescence in situ hybridization, *HER2* amplification, HER2 immunohistochemistry, *HER2*-positive breast carcinoma, Next-generation sequencing

## Abstract

**Background:**

Human epidermal growth factor receptor 2 *(HER2*) positive breast carcinomas due to *HER2* amplification are associated with aggressive behavior and a poor prognosis. Anti-HER2-targeted therapies are widely used to treat *HER2*-positive breast carcinomas with excellent outcomes. Accurate identification of HER2 amplification status in breast carcinomas is of important diagnostic and treatment value. Currently, HER2 amplification status is routinely determined by immunohistochemistry (IHC) and/or fluorescence in situ hybridization (FISH) testing. This study will review our past HER2 data to determine and characterize discordant results between HER2 IHC and FISH. It will also determine a potential impact of *HER2* amplification status by next-generation sequencing (NGS) on these patients.

**Methods:**

We reviewed a total of 4884 breast carcinomas with coexisting HER2 IHC and *HER2* FISH performed at our institution from 2010 to 2022. 57 cases also had a Next-Generation-Sequencing-based (NGS) gene panel performed. Given the advances in biostatic analysis pipelines, NGS methods were utilized to provide results on *HER2* amplification status along with somatic mutations.

**Results:**

While the majority (ranging from 98.5% with IHC score of 0 and 93.1% with IHC score of 1 +) of 4884 breast carcinomas had concordant results from HER2 IHC and *HER2* FISH testing, a small percentage of patients (ranging from 1.5% in those with IHC score of 0, to 6.9% with IHC score of 1 +) had discordant results, with negative HER2 IHC and positive *HER2* FISH results. These patients could be reported as *HER2*-negative breast carcinomas if only HER2 IHC testing has been performed according to a current cost-effective HER2 test strategy. 57 patients had *HER2* amplification status determined by NGS, and all patients had concordant results between *HER2* NGS and FISH tests. A *HER2*-amplified breast carcinoma by NGS had a negative IHC and a positive *HER2* FISH result. This case was classified as a *HER2*-positive breast carcinoma, had anti-HER2-targeted therapy, and achieved a complete clinical response.

**Conclusions:**

A small percentage of *HER2*-positive breast carcinomas are unidentified because of a negative HER2 IHC based on our current cost-effective HER2 test strategy. It is not feasible and affordable in routine clinical practice to perform *HER2* FISH for the cases with negative HER2 IHC (IHC score 0 and 1 +). Therefore, NGS assays capable of simultaneously detecting both somatic mutations and *HER2* amplification could provide a more comprehensive genetic profiling for breast carcinomas in a clinical setting. Identification of *HER2* amplification by NGS in *HER2*-positive breast carcinomas with negative HER2 IHC results is important since these cases are concealed by our current cost-effective HER2 test strategy with IHC first (for all cases) and FISH reflex (only for cases with IHC score of 2 +), and would offer the opportunity for potentially beneficial anti-HER2-targeted therapies for these patients.

## Background

Breast cancer is the most common cancer and second leading cause of death among all cancers in women [1] and is a heterogeneous disease [2]. Human epidermal growth factor receptor 2 (*HER2*) due to amplification of its coding gene has been described in approximately one fifth of primary invasive breast carcinomas [3–5]. *HER2* amplified (*HER2*-positive) breast carcinomas are associated with aggressive behavior and a poor prognosis compared with those in which *HER2* is not amplified [4]. Anti-HER2-targeted therapies are widely used to treat HER2-positive breast carcinomas with excellent outcomes and have no role in the treatment of HER2-negative breast carcinomas [6–9].

Adjuvant Anti-HER2-targeted therapies significantly improve outcomes for patients with HER2-positive early breast cancer. In HER2-positive early breast cancer, anti-HER2 therapy together with neoadjuvant chemotherapy has become the standard of care as achievement of pathological complete response is correlated with improved progression-free survival and disease-free survival [10]. In the neoadjuvant setting, dual HER2-blockade with trastuzumab and pertuzumab together with chemotherapy improves rates of pathological complete response and is, therefore, considered standard of care [11]. In patients with HER2-positive metastatic breast cancer, progression-free survival and overall survival were significantly improved and maintained after first-line therapy with anti-HER2 monoclonal antibodies pertuzumab and trastuzumab in combination together with docetaxel (pertuzumab group) [12, 13]. HER2-targeted therapy has dramatically changed the natural history of HER2-positive metastatic breast cancer, and the recommended first-line therapy for HER2-positive metastatic breast cancer consists of the anti-HER2 monoclonal antibodies trastuzumab and pertuzumab [14]. Therefore, accurate identification of *HER2* amplification status in breast carcinomas is of important diagnostic and treatment value and is necessary to ensure adequate patient treatment management, better treatment planning, and avoid patient exposure to unnecessary and potentially harmful treatments.

All patients undergo HER2 testing upon primary breast cancer diagnosis, relapsed and metastatic setting to inform treatment decisions [15]. HER2 amplification status is routinely determined by immunohistochemistry (IHC) and/or fluorescence in situ hybridization (FISH) testing [16]. The HER2 IHC testing indirectly measures overexpression of HER2 receptors on the surface of breast cancer cells, based on the intensity of the color reaction. HER2 protein expression status by IHC ranges from 0 to 3 + , with HER2 IHC scores of 0 reported as HER2-negative breast carcinomas, and HER2 IHC score of 3 + reported as HER2-positive breast carcinomas [17]. Currently, breast cancer with HER2 IHC score of 1 + or 2 + and negative FISH result defines as HER2-low breast cancer [18, 19]. *HER2* FISH testing measures the exact number of copies of the *HER2* gene per nucleus and the ratio between the *HER2* gene and a control probe to determine *HER2* amplification status. Both the HER2 IHC and FISH testing are US Food and Drug Administration (FDA) approved methods for the determination of HER2 amplification status. As a routine practice for all newly diagnosed breast cancer in pathology, the HER2 test strategy with a low cost-effectiveness ratio involves screening all newly diagnosed breast cancers with IHC testing (a screening test), and reflexing HER2 IHC score of 2 + for evaluation by FISH testing (as a confirmation/follow-up test) [17, 20]. Interpretation of HER2 IHC and *HER2* FISH test results include five different groups for breast carcinomas with HER2 IHC score of 2 + further evaluated by *HER2* FISH testing [21–23]. *HER2* FISH testing is usually not performed for invasive breast carcinomas with IHC score of 0 or 1 + and positive (IHC 3 +) HER2 IHC testing results [21–23]. Although HER2 IHC and FISH testing are mostly concordant, a small percentage of patients (~ 1.5%) have been described to have discordant HER2 IHC and FISH results with negative HER2 IHC and positive *HER2* FISH results [24]. These patients showed either complete remission or partial remission after anti-HER2-targeted therapies [24]. Somatic DNA mutations in invasive breast carcinomas are important [25] and could be commonly detected by next-generation sequencing (NGS) methods. Given the advances in biostatic analysis pipelines, NGS DNA data could be utilized to provide results on both somatic mutations and copy number alterations (such as gain, amplification, loss) in the same assay [26, 27]. NGS data could be used to confirm *HER2* amplification in *HER2*-positive invasive breast carcinomas with positive HER2 IHC results and to reveal *HER2* amplification in *HER2*-positive invasive breast carcinomas with negative HER2 IHC results.

Here, we performed a retrospective review of concurrent HER2 IHC and *HER2* FISH performed at our institution over the past 12 years to determine the frequency of breast carcinomas with discordant results between HER2 IHC and FISH, such as negative HER2 IHC and positive *HER2* FISH results. These breast carcinomas would be reported as *HER2*-negative breast carcinomas according to negative HER2 IHC results if only IHC had been performed. Given the advances in biostatic analysis pipelines, NGS methods detect not only somatic mutations, but also have been utilized to evaluate *HER2* amplification status. 57 breast carcinoma specimens had NGS data for somatic mutations and *HER2* amplification status determined using NGS copy number alteration pipelines [28]. *HER2* amplification status was further compared between *HER2* NGS and FISH testing to determine whether *HER2* amplification status by NGS could be useful to reveal a subset of *HER2*-positive breast carcinomas with a negative IHC and a positive *HER2* FISH result. Identification of *HER2* amplification by NGS in *HER2*-positive breast carcinomas with negative HER2 IHC results is important to reveal those cases that are hidden by our current HER2 test strategy with IHC first (for all cases) and FISH reflex (only for cases with IHC score of 2 +).

## Methods

### Patients and samples

A clinical database was maintained for breast carcinomas that had coexisting HER2 IHC and *HER2* FISH results as part of routine clinical testing from January 1, 2010 to April 30, 2022. We identified a study cohort (n = 4884 cases) with breast carcinoma as a general diagnosis. A subset (n = 57) of breast carcinoma specimens in this study cohort also had *HER2* amplification status determined by NGS. The institutional review board of the hospital approved this study (JHIRB00339499).

### HER2 immunohistochemistry (HER2 IHC)

HER2 immunohistochemistry was performed on the formalin fixed paraffin embedded (FFPE) breast carcinoma specimens and was analyzed with the Ventana PATHWAY system, using an anti-HER2/neu (4B5) rabbit monoclonal primary antibody and Ventana iVIEW DAB Detection Kit (Ventana, Tucson, Arizona, USA). Positive HER2 (IHC score of 3 +) was defined as intense, complete, and circumferential membrane staining in more than 10% of invasive tumor cells. Equivocal HER2 expression (IHC score of 2 +) was defined as weak to moderate complete membrane staining observed in more than 10% of tumor cells, and/or intense complete membrane staining in less than 10% of tumor cells. IHC score of 2–3 + focal was defined as heterogeneous tumor population with some IHC 2 + regions and some IHC 3 + regions. HER2-negative status was defined as either incomplete/faint membrane staining in more than 10% of invasive tumor cells (IHC score of 1 +) or no staining or incomplete/faint membrane staining in less than 10% of invasive tumor cells (IHC score of 0). IHC score of 1–2 + focal was defined as heterogeneous tumor population with some IHC 1 + regions and some IHC 2 + regions. For our institutional standard practice, HER2 IHC was performed on all breast carcinoma cases and primarily the equivocal IHC (sore of 2 +) cases were reflexed to perform *HER2* FISH. Occasionally, a subset of breast carcinomas had concurrent HER2 IHC and *HER2* FISH tests per clinical requests.

### *HER2* fluorescence in situ hybridization (*HER2* FISH)

*HER2* gene amplification by FISH was tested using a *HER2* FISH probe set according to the manufacturer’s recommendations (PathVysion *HER2* DNA Probe Kit, Vysis, Abbott Molecular, Des Plaines, Illinois, USA). A total of 60 nuclei were visually evaluated with fluorescence microscopy by two technologists scoring blinded from each other using a Zeiss Axioscope system (Carl Zeiss Microscopy, LLC, White Plains, NY, USA). The analysis was performed using Cytovision software version 7.7 (Leica Inc., Buffalo Grove, IL, USA). *HER2* FISH results were classified according to the 2018 ASCO/CAP guideline into 5 groups/categories. Groups 1 and 3 are reported as *HER2*-positive breast carcinomas, and group 5 is reported as *HER2*-negative breast carcinoma [23]. For groups 2 and 4, an additional 20 nuclei were evaluated by a third blinded technologist and were reported as *HER2*-negative breast carcinomas for HER2 IHC scores of 2 + [23]. Depending on the date of testing, *HER2* FISH results were reclassified according to the 2018 ASCO/CAP guideline [23]. HER2 IHC and *HER2* FISH tests were performed on the same tumor block(s) from the same specimen.

### *HER2* copy number estimation by a NGS assay

The target NGS assay was described previously [28, 29]. Briefly, DNA was extracted from FFPE specimens with the Siemens tissue preparation automated method (Siemens Healthineers, Munich, Germany). DNA concentration was assessed by the Qubit fluorometer according to vendor specification (Thermo Fisher Scientific, Waltham, MA, USA). Library preparation was performed using Kapa Roche HyperPrep reagents (Roche Diagnostics, Inc., Wilmington, MA, USA), and hybrid capture was executed using 40,670 Integrated DNA Technologies probes (Integrated DNA Technologies, Inc., Coralville, IA, USA). Copy number alterations from the NGS data generated using bioinformatics pipelines were described previously [28, 30]. NGS coverage-based copy number estimation based on the sequencing coverage depth was used to calculate Log2 fold change for copy number detection [28]. Log2 ratio thresholds were set at ≥ 1.3 as a positive result for *HER2* amplification and at < 1.3 as a negative result for *HER2* amplification. *HER2* amplification status by NGS has been adopted clinically since last year.

### Statistical calculators

Comparison of numerical variables was performed by the Chi-square calculator and Fisher exact test calculator (Social Science Statistics, https://www.socscistatistics.com, last accessed on July 6, 2022). *P* ≤ 0.05 was considered statistically significant. Comparison of sensitivity, specificity, positive predictive value, negative predictive value, and the accuracy of the *HER2* amplification status by NGS and FISH methods was performed using MEDCALC statistical software (https://www.medcalc.org/calc/diagnostic_test.php, last accessed July 6, 2022).

## Results

### Retrospective review of concurrent HER2 IHC and *HER2* FISH

**Fig. 1:**
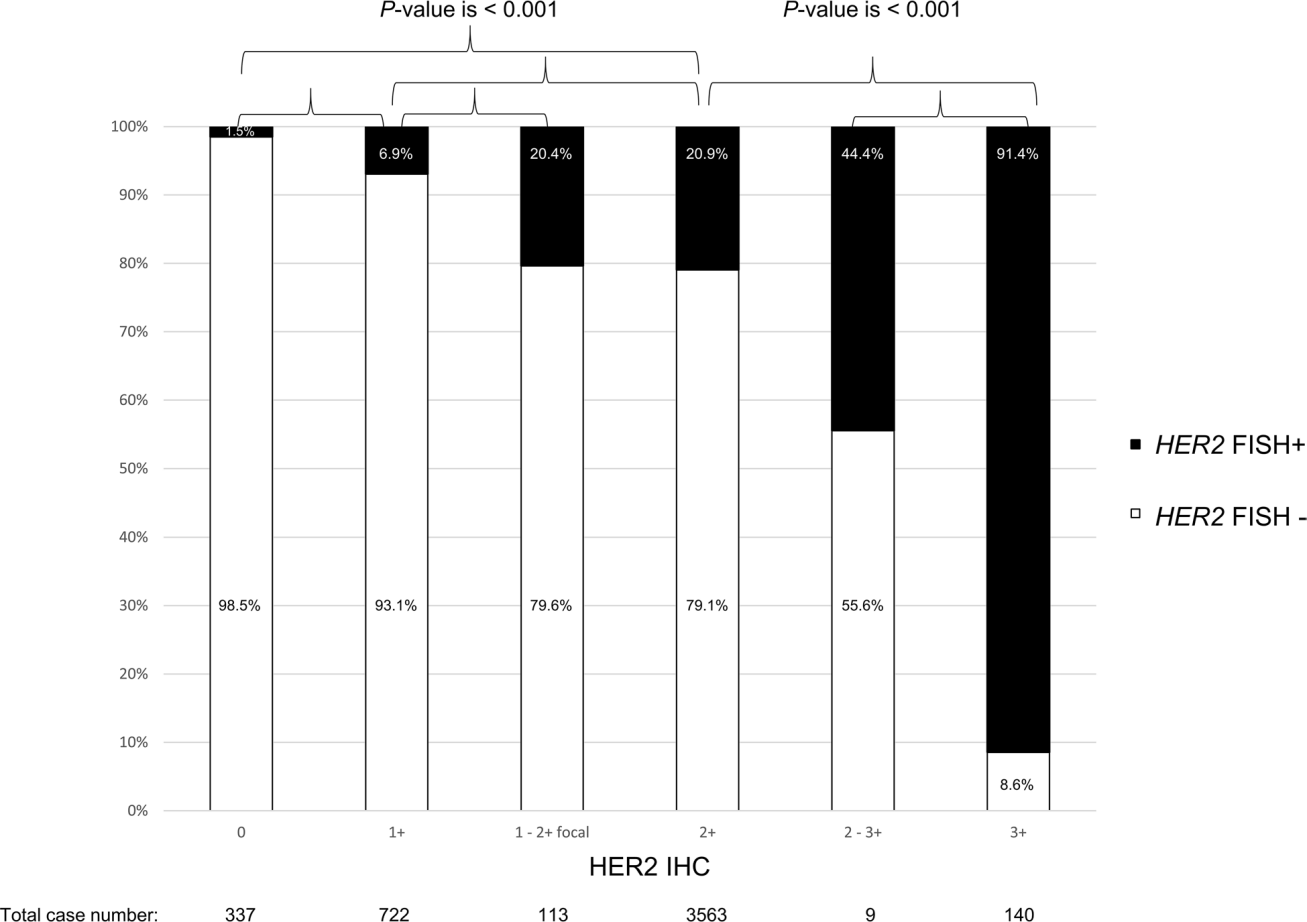
Percentages of *HER2* FISH in different HER2 IHC groups (scores range from 0 to 3 +). HER2 IHC negative (score of 0) had the lowest percentage (1.5%), and HER2 IHC positive (score of 3 +) had the highest percentage (91.4%)

Among 4884 breast carcinomas that had concurrent HER2 IHC and *HER2* FISH, over one-fifth of cases (n = 1059, 21.7%) had a negative HER2 IHC result (IHC score of 0 and 1 +) and approximate three-fourth of cases (n = 3563, 73.0%) had an equivocal IHC result (IHC score of 2 +) (Fig. 1). Positive *HER2* FISH was detected at 1.5, 6.9, 20.4, 20.9, 44.4, and 91.4% in cases with IHC scores of 0, 1 + , 1–2 + focal, 2 + , 2–3 + focal, and 3 + , respectively (Fig. 1). FISH positivity in the IHC score of the 1 + group was significantly higher than in the IHC score of the 0 group (P < 0.001) (Fig. 1). The IHC 2 + group had a significantly higher FISH positivity compared to negative IHC (score of 0 and 1 +) groups (P < 0.001) (Fig. 1). No significantly different positivity of FISH was observed among IHC scores of 1–2 + focal, 2 + , and 2–3 + focal groups (Fig. 1).

### HER2 amplification status by NGS, FISH, and IHC

Among 57 breast carcinomas analyzed by NGS, *HER2* FISH, and IHC, 3 (5.3%) had *HER2* amplification by NGS, all of which had positive *HER2* FISH results. 54 (94.7%) were negative for *HER2* amplification by NGS, all of which had negative *HER2* FISH results. Therefore, the sensitivity, specificity, positive predictive value, negative predictive value, and accuracy of the *HER2* amplification status by NGS were 100% compared with *HER2* FISH.

Of three breast carcinomas with *HER2* amplification by NGS, one (33.3%) had a negative HER2 IHC (score of 1 +) and two (66.7%) had a positive HER2 IHC (score of 3 +). The remaining 54 breast carcinomas had either negative or equivocal HER2 IHC (score of 0–2 +). Therefore, the sensitivity, specificity, positive predictive value, negative predictive value, and accuracy of the *HER2* amplification status by NGS were 100%, 98.2%, 66.7%, 100%, 98.3%, respectively, compared with *HER2* IHC.

### A *HER2*-positive breast carcinoma by NGS with negative HER2 IHC

**Fig. 2:**
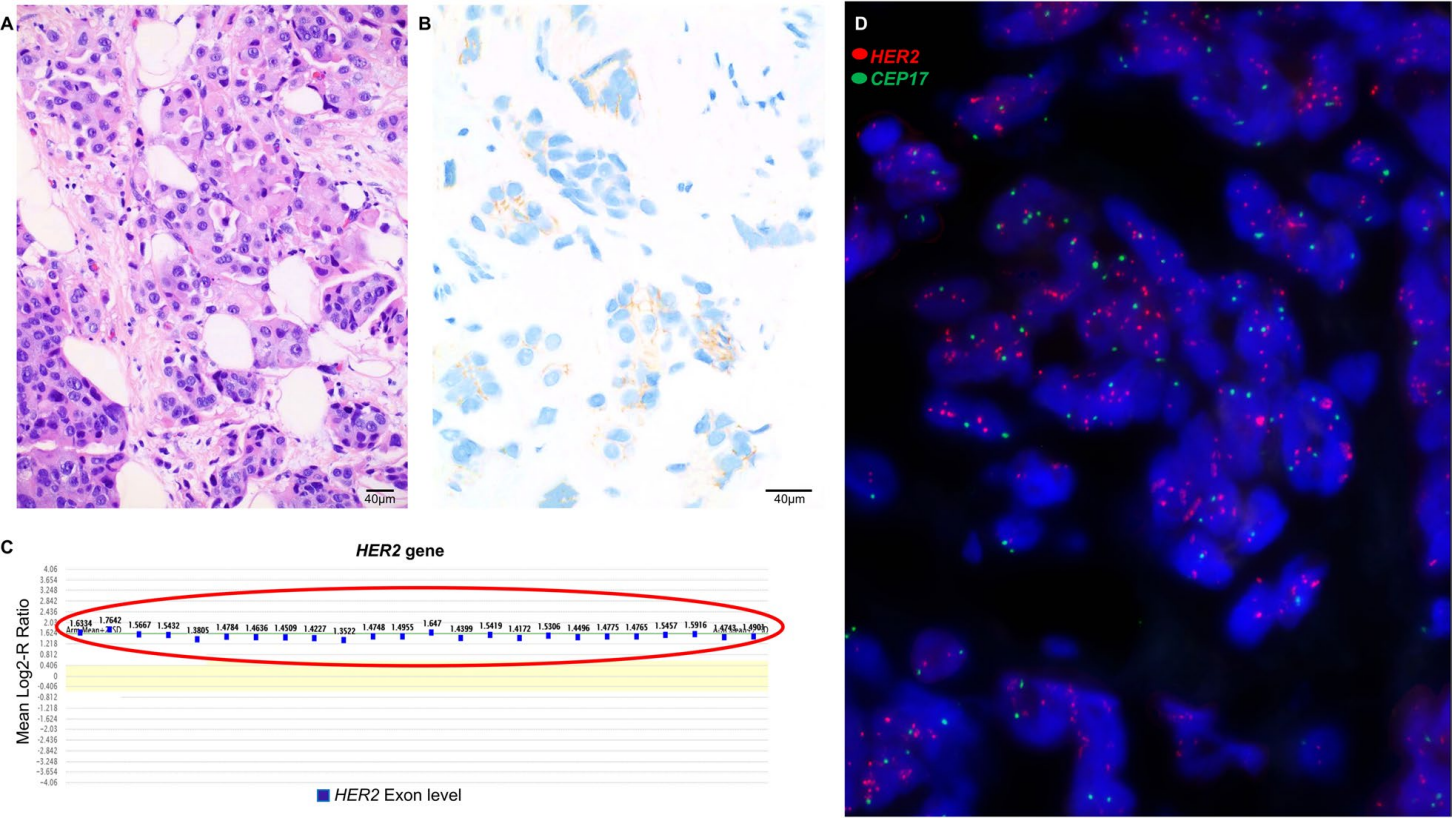
*HER2* amplification by NGS in a breast carcinoma with a negative HER2 IHC result. **A**, **B** Microscopy image of HER2 IHC 1 + in a surgical specimen. **A** is hematoxylin and eosin (H & E) staining and **B** is HER2 IHC staining. **C** Amplification of all probes of the *HER2* gene by next-generation sequencing based on Log2-R ratio (> 1.3). **D** *HER2* amplification by FISH. Dual-color *HER2* FISH revealed ratio of the *HER2* to D17Z1 (a centromere control probe) = 4.0, average number of *HER2* signals per nucleus = 8.4, and average number of *CEP17* signals per nucleus = 2.1

A brief clinical presentation of a *HER2* amplified breast carcinoma by NGS with negative HER2 IHC follows. A 33 year-old female patient had left breast invasive ductal carcinoma. HER2 IHC testing was negative (score of 1 +) and reported as a HER2-negative breast carcinoma (Fig. 2A, B). IHC also revealed positive estrogen (ER) and negative progesterone (PR) results (data not shown). NGS revealed a pathogenic *GATA3* frame-shift mutation, which occurs in approximately 15% of primary ER positive breast carcinomas [25, 31]. No other mutations such as *BRCA1, BRCA2, PIK3CA,* and *ESR1* genes were observed. However, *HER2* amplification was detected by NGS based on Log2-R ratio (Fig. 2C). *HER2* FISH confirmed the *HER2* amplification (Fig. 2D) in this specimen as a group 1 positive *HER2* FISH result per 2018 ASCO/CAP breast cancer guideline for dual color probes when the *HER2*/CEP17 ratio is ≥ 2 and the average *HER2* copy number is ≥ 4.0 signals per cell [23]*.* Positive *HER2* FISH results with or without HER2 proteins by IHC are considered *HER2*-positive breast carcinomas. The patient was then treated with anti-HER2-targeted therapy and showed a complete clinical response.

## Discussion

In our current laboratory practice, testing for HER2 expression by immunochemistry is routinely performed for all breast carcinomas. HER2 IHC is one of the most rigorously controlled techniques, and guidelines for testing standardization, specimen handling, and reporting were established to guarantee accuracy and decrease laboratorial variability. Although the concordance rate between HER2 IHC and FISH is very high, a small percentage of patients had discordant results with negative HER2 IHC and positive *HER2* FISH results: 1.5% positive *HER2* FISH in patients with HER2 IHC score of 0, and 6.9% positive *HER2* FISH in patients with HER2 IHC score of 1 in this study cohort of 4884 breast carcinomas. Given the significantly high positivity of FISH in the IHC score of 1 + group compared to the IHC score of 0 group in this study, it is important to distinguish between these 2 groups. Although IHC 1 + and IHC 0 groups may have different pathological and clinical features [32, 33], there could be challenges to achieve a good concordance rate by IHC among US laboratories [34].

Several technical and non-technical factors may contribute to the negative IHC and positive FISH breast carcinoma phenomenon. Besides pre-analytic technical issues, such as HER2 IHC signal intensity decreasing over time [35], and IHC performed on old sections stored for more than 6 weeks [23], other biologic factors including intra-tumor genomic heterogeneity, co-amplification/polysomy 17 and monosomy 17 may contribute to this phenomenon [32, 36–43]. HER2 intra-tumor genomic heterogeneity is the co-existence of multiple tumor cell populations with discernibly different levels of HER2 expression within the same tumor, which has been reported in up to half of breast cancers [32, 41–43]. Aneuploidy of chromosome 17, including gain (trisomy/polysomy 17) and loss (monosomy 17), has been reported in breast carcinomas [36–39]. HER2 intra-tumor genomic heterogeneity along with aneuploidy chromosome 17 may further lead to skewing IHC results [41]. Chromosomal microarray or alternative probes on chromosome 17 are required to distinguish between true polysomy 17 and co-amplification/focal amplification [44, 45].

Because of low cost and advances in NGS technology, the NGS gene panel is clinically adopted to be used to identify actionable mutations for targeted therapy. Besides HER2-targeted therapy significantly improving the survival of HER2-positive breast cancer patients, other targeted therapies are also a powerful therapeutic strategy for breast cancer, such as poly (ADP-ribose) polymerase inhibitors (olaparib and talazoparib) for BRCA1/2 mutation carriers [46], PI3K-alpha inhibitors (alpelisib) and estrogen receptor antagonist (fulvestrant) for *PIK3CA* activating mutations [47], tumor-agnostic tropomyosin receptor kinase inhibitors (larotrectinib and entrectinib) for *NTRK* fusions [48], immune checkpoint inhibitors (pembrolizumab) for high tumor mutation burden [49], etc. Given progressive biostatic analysis pipelines, the NGS assay is utilized to simultaneously detect both copy number variants and somatic mutations, which could provide more comprehensive genetic profiling for cancer patients using a single assay in a clinical setting. In our current NGS cohort with breast carcinomas, *HER2* amplification status by NGS achieves a concordant result compared to *HER2* FISH. NGS also identified a *HER2* amplified breast carcinoma with a negative HER2 IHC, which could go unidentified by the current HER2 IHC-first test approach. HER2 IHC negative cases are reported as HER2-negative breast carcinomas without follow-up FISH in the majority of US laboratories. Concurrent HER2 IHC and FISH tests are not commonly used because of the high cost and complexity requirements of the FISH testing. This cost-effective test approach will miss a small percentage of breast carcinomas that have discordant HER2 IHC and FISH results.

**Fig. 3:**
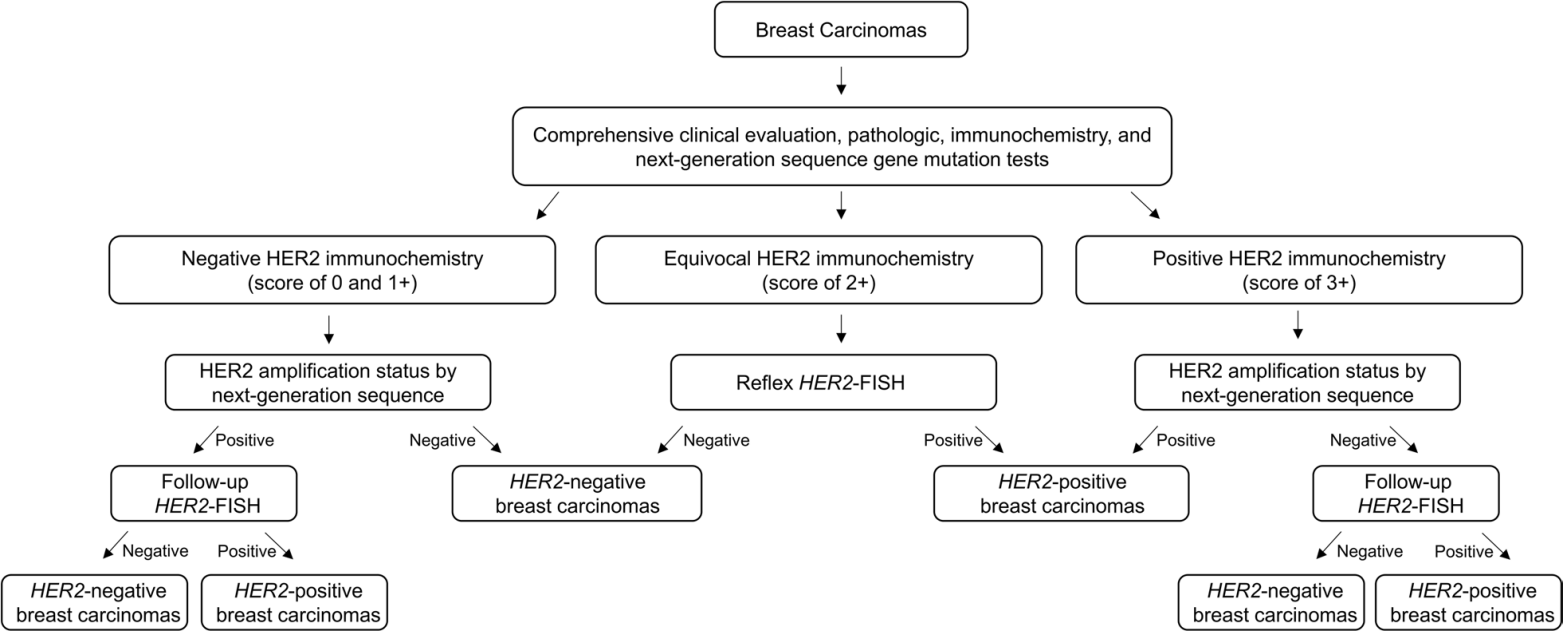
A combination of HER2 IHC and *HER2* amplification status by NGS sequence in a cost-effective HER2 test strategy

The current study suggests potential clinical utilities of *HER2* amplification status by NGS in a cost-effective HER2 test strategy (Fig. 3). Currently, NGS gene panel is used commonly to identify targetable biomarkers. For breast carcinomas, HER2 IHC and NGS are commonly performed. We outlined an algorithm for the *HER2* amplification workup in breast carcinomas based on a common HER2 IHC first approach (Fig. 3). Based on HER2 IHC results, breast carcinomas can be categorized as HER2-negative, equivocal, or positive breast carcinomas (the second decision branch in Fig. 3). Equivocal HER2 IHC (score of 2 +) will automatically reflex to *HER2* FISH to further determine *HER2* amplification status. For IHC negative and positive breast carcinomas, *HER2* amplification status by NGS is recommended to further determine *HER2* amplification categories (the third decision branch in Fig. 3). Follow-up *HER2* FISH will only be performed for the cases with negative IHC/positive NGS and positive IHC/negative NGS (the fourth decision branch in Fig. 3). This approach dramatically reduces the follow-up *HER2* FISH number, since discordant IHC/FISH is only present in a small percentage of IHC scores of 0–1 + and 3 + in this study. Using a combination of HER2 IHC and NGS could achieve the optimum balance of sensitivity and specificity to identify *HER2* amplified breast carcinomas among discordant HER2 IHC and FISH results. Identification of *HER2* amplified breast carcinomas with negative IHC results is important and might provide potentially beneficial anti-HER2-targeted therapies for these patients. Although NGS mutation pipelines have been widely adopted into clinical labs, NGS copy number alteration pipelines have not been commonly established across all NGS panels. This proposed algorithm has a crucial limitation since not all NGS panels provide information in copy number alterations. However, given further advances in NGS copy number alteration pipelines throughout all NGS panels, this algorithm might become more integrated into the routine diagnostic workflow of clinical labs.

## Conclusion

*HER2*-positive invasive breast carcinomas with negative HER2 IHC results and positive *HER2* FISH results are usually concealed by our current *HER2* test strategy with HER2 IHC testing as a screening test for all newly diagnosed breast cancer. Given the advances in biostatic analysis pipelines, NGS-based methods could be utilized to provide results on both somatic mutations and copy number alterations (such as *HER2* amplification) in the same assay. Identification of *HER2* amplification by NGS in *HER2*-positive invasive breast carcinomas with negative HER2 IHC results would provide the opportunity for potentially beneficial anti–HER2-targeted therapies. Further research is needed to understand the mechanisms of *HER2* amplification without detectable HER2 proteins, as well as the exact mechanism by which HER2 antibody–drug conjugates are the most effective in these patients.

## Data Availability

The dataset for the current study is available from the corresponding author on reasonable request.
